# The Functional Implications of Broad Spectrum Bioactive Compounds Targeting RNA-Dependent RNA Polymerase (RdRp) in the Context of the COVID-19 Pandemic

**DOI:** 10.3390/v15122316

**Published:** 2023-11-25

**Authors:** Brittany A. Comunale, Robin J. Larson, Erin Jackson-Ward, Aditi Singh, Frances L. Koback, Lilly D. Engineer

**Affiliations:** 1 Department of Health Policy and Management, Johns Hopkins Bloomberg School of Public Health, Johns Hopkins University, Baltimore, MD 21205, USA; 2 Geisel School of Medicine at Dartmouth, Hanover, NH 03755, USA; 3 Department of Palliative Medicine, Dartmouth-Hitchcock Medical Center, Lebanon, NH 03756, USA; 4 Cedars-Sinai Medical Center, Los Angeles, CA 90048, USA; 5 Department of Biological Sciences, University of California San Diego, La Jolla, CA 92161, USA; 6 Department of Anesthesiology and Critical Care Medicine, Johns Hopkins School of Medicine, Baltimore, MD 21205, USA

**Keywords:** NSP12 protein, SARS-CoV-2, coronavirus RNA-dependent RNA, polymerase, scoping review, RNA synthesis inhibitors, drug repurposing, antiviral agents

## Abstract

Background: As long as COVID-19 endures, viral surface proteins will keep changing and new viral strains will emerge, rendering prior vaccines and treatments decreasingly effective. To provide durable targets for preventive and therapeutic agents, there is increasing interest in slowly mutating viral proteins, including non-surface proteins like RdRp. Methods: A scoping review of studies was conducted describing RdRp in the context of COVID-19 through MEDLINE/PubMed and EMBASE. An iterative approach was used with input from content experts and three independent reviewers, focused on studies related to either RdRp activity inhibition or RdRp mechanisms against SARS-CoV-2. Results: Of the 205 records screened, 43 studies were included in the review. Twenty-five evaluated RdRp activity inhibition, and eighteen described RdRp mechanisms of existing drugs or compounds against SARS-CoV-2. In silico experiments suggested that RdRp inhibitors developed for other RNA viruses may be effective in disrupting SARS-CoV-2 replication, indicating a possible reduction of disease progression from current and future variants. In vitro, in vivo, and human clinical trial studies were largely consistent with these findings. Conclusions: Future risk mitigation and treatment strategies against forthcoming SARS-CoV-2 variants should consider targeting RdRp proteins instead of surface proteins.

## 1. Introduction

The severe acute respiratory syndrome coronavirus 2 (SARS-CoV-2) (coronavirus disease (COVID-19)) pandemic has had devastating effects in countries around the world for the past 3 years and continues to incite uncertainty about the transmissibility and virulence of newly emerging variants [1]. Such concern is warranted, as RNA viruses have a well-established proclivity to gradually develop genetic point mutations that result in changes in their surface proteins, and SARS-CoV-2 is no exception [2]. This process, termed antigenic drift, describes how a virus can drift from its original form, engender a new viral strain, and potentially prevent immune systems from recognizing the new proteins [3]. Thus, circulating strains can evolve in a way that makes available vaccines, which were originally created to target the surface proteins of a preceding strain, ineffective [4]. While ongoing clinical trials are actively developing and testing COVID-19 vaccines against SARS-CoV-2 strains that have recently appeared, new strains are emerging more rapidly than vaccines are being developed. Additionally, access to vaccines and COVID-19 treatments are not equitable around the world, and many populations lack protective measures against both current and future variants of concern [5].

As prophylactic and therapeutic agents that target surface proteins leave recipients susceptible to infection by new SARS-CoV-2 strains, there is a need to find drugs and compounds that target viral proteins and are less likely to mutate.

One such protein of interest is the RNA-dependent RNA polymerase (RdRp) protein. In contrast to a surface protein, such as the spike (S) or main protease (Mpro), RdRp is a core protein found in most RNA viruses that is mainly responsible for viral replication or RNA synthesis [6]. In the case of SARS-CoV-2, the RdRp protein, also known as nonstructural protein 12 (nsp12), efficiently generates RNA template strands for replication once it has formed a complex with other nonstructural proteins, particularly nsp7 and nsp8 [7,8]. Given the energy-intensive nature of RNA synthesis, adenosine triphosphate (ATP), the major molecule used to store and transfer energy in cells, is required to maintain replication processes [9,10]. When ATP enters the RdRp cavity and interacts with the catalytic site, it prompts the formation of new bonds between ribonucleotides and elongates RNA strands [11]. Thus, viral replication, which leads to disease progression, relies on RdRp functionality. 

RdRp is considered to be highly conserved, or relatively unchanged in an evolutionary sense, due to structural and functional similarities across viruses, such as SARS-CoV, hepatitis C, poliovirus, Ebola virus, Zika virus, dengue virus, and yellow fever [12,13,14,15]. Accordingly, researchers have been interested in whether drugs that were initially developed for another RNA virus could be repurposed against SARS-CoV-2. The most well-known therapeutic of this type is remdesivir, an RdRp inhibitor that was originally produced to treat the Ebola virus and is now commonly used to mitigate the severity of critical SARS-CoV-2 cases [16,17,18]. Since remdesivir can only be administered intravenously, its use is restricted to hospitals, which not only excludes populations that have geographic, financial, or social access barriers but also prevents aid among those who may not seek services quickly enough. 

In addition to remdesivir, other RdRp inhibitors developed for different RNA viruses may also be useful in combatting SARS-CoV-2, particularly with imminent variant strains, due to the RdRp protein being less likely to mutate compared to the spike protein. Analyzing SARS-CoV-2 mutation rates across the genome, researchers have found that the spike protein is largely responsible for emerging variants, with the highest rate of mutations (13.5 ± 0.4 × 10^−6^ nt^−1^/cycle^−1^ mutations in the CoV-2-G genotype and 17.1 ± 1.0 × 10^−6^ in the CoV-2-D genotype) at five times the genomic averages. In contrast, the accumulation of mutations for the RdRp protein is about 1.28 × 10^−6^ nt^−1^/cycle^−1^ mutations in the CoV-2-G genotype and 1.38 × 10^−6^ in the CoV-2-D genotype [19]. While some RdRp inhibitors may be more well known than others, a broad awareness of which existing drugs and compounds are currently being considered for repurposing, as well as an understanding of why these agents may be effective against SARS-CoV-2, could have significant implications for future practices, policies, and research. 

The aim of this scoping review was to define the scope of published data describing RdRp inhibitors in the context of the COVID-19 pandemic, with specific interest in functional implications for future therapeutic targets. To the best of our knowledge, no reviews have been conducted to identify which or why existing RdRp inhibitors may be useful in diminishing the risk of SARS-CoV-2 infection and disease progression or to understand the mechanisms by which RdRp inhibitors may disrupt replication. While there is evidence to suggest that available drugs may inhibit SARS-CoV-2 RdRp based on in silico studies (i.e., computer simulations), an understanding based on in vitro (i.e., laboratory experiments performed outside of living organisms, such as in a petri dish or cell culture wells), in vivo (studies performed on living organisms, such as mice or monkeys), and human trial studies is also required to further validate these claims [20,21,22,23]. Thus, there is a need to synthesize the available information of in silico, in vitro, in vivo, and human trial data that identify potential RdRp inhibitors; explore the possible underlying mechanisms; and assess whether predicted RdRp activities in silico may be validated by other methods. Such potential insights may inform risk mitigation and treatment strategies against future SARS-CoV-2 variants. 

## 2. Materials and Methods

The Preferred Reporting Items for Systematic Reviews and Meta-Analyses extension for scoping reviews (PRISMA-ScR) criteria and recommendations listed in the PRISMA 2020 Statement informed the methodology of this scoping review [24,25].

### 2.1. Data Sources and Search Strategy

Under the guidance of a biomedical public health specialist librarian, we searched MEDLINE/PubMed and EMBASE to identify studies that explored the relationship between RdRp and COVID-19. First, we created a set aimed at capturing these high-level concepts by using the Boolean term “AND” to combine medical subject heading (MeSH) terms and keywords related to RNA-dependent RNA polymerase and SARS-CoV-2. Next, to capture the studies aimed at exploring the functional implications of RdRp in the context of the COVID-19 pandemic, we created a set that “OR’ed” key terms related to inhibition. Finally, we created a set that “OR’ed” key terms related to molecular structures, mechanisms, and variants. The intention of the last set was to focus the review on studies that aimed to understand why existing compounds or RdRp inhibitors, which were not specifically developed to combat SARS-CoV-2, may be used against the novel coronavirus. For instance, are the RdRp structures across viruses so similar that inhibitors made for one could be used against another? Is the underlying mechanism to disrupt viral replication the same for all RdRp inhibitors? If an RdRp inhibitor is effective against one virus, could it also inhibit the viral replication of SARS-CoV-2, not only for the current strains but also in emerging variants? Finally, we used the Boolean term “AND” to find the intersection of the four sets. Publication dates were restricted from 2019 to the present to exclude anything written prior to the COVID-19 pandemic.

MeSH terms and closely related keywords were included in the MEDLINE/PubMed database search to ensure study comprehensiveness and reduce the likelihood of omitting pertinent articles. Reference lists from relevant studies were also examined to supplement the computerized literature search.

The following search strategy was used in MEDLINE/PubMed: 

(“RNA-Dependent RNA Polymerase” (MeSH) OR “RNA Dependent RNA Polymerase” (tw) OR “RdRp protein” (tw) OR “RNA dependent RNA replicase” (tw) OR “RNA directed RNA replicase” (tw) OR “RNA replicase” (tw)) AND (“Coronavirus Infections” (MeSH:NoExp) OR “SARS-CoV-2” (MeSH) OR “COVID-19” (MeSH) OR “SARS-CoV-2” (tw) OR “COVID-19” (tw) OR “Coronavirus Infection*” (tw)) AND (inhibit (tw) OR inhibition (tw) OR inhibitor (tw)) AND (mechanism* (tw) OR structure (tw) OR variant* (tw)) 

An adapted strategy was used in EMBASE: Concept #1 AND Concept #2 AND Concept #3 AND Concept #4

- Concept #1: ‘RNA directed RNA polymerase’/exp OR (‘RNA-dependent RNA polymerase’ OR ‘RNA dependent RNA replicase’ OR ‘RNA directed RNA replicase’ OR ‘RNA replicase’):ab,ti,kw
- Concept #2: ‘coronavirus disease 2019’/exp OR (‘2019-nCoV disease’ OR ‘COVID 19’ OR ‘SARS-CoV-2 disease’ OR ‘SARS-CoV-2 infection’ OR ‘SARS-CoV-2 pneumonia’ OR ‘SARS-CoV2 disease’ OR ‘SARS-CoV2 infection’ OR ‘SARSCoV2 disease’):ab,ti,kw
- Concept #3: (‘inhibit’ OR ‘inhibition’ OR ‘inhibitor’):ab,ti,kw
- Concept #4: (‘mechanism’ OR ‘variant’ OR ‘structure’):ab,ti,kw

### 2.2. Eligibility Criteria and Study Selection

Two reviewers (B.A.C. and R.J.L.) determined the initial eligibility criteria by consensus a priori. Records were imported into EndNote 20, and duplicate records were removed. Abstracts were then screened (B.A.C.) according to the defined inclusion and exclusion parameters. 

Records were considered eligible if (1) it was a study that included original, empirical data; (2) it focused on the relationship between RdRp and the inhibition of RNA synthesis or viral replication; and (3) RdRp inhibition was analyzed in the context of the COVID-19 pandemic. We excluded studies if (1) we were not able to obtain the full text or (2) it was in a language for which we could not obtain a translation. No records were excluded due to language barriers.

During the process of screening studies, four primary themes emerged based on each article’s main focus: RdRp activity inhibition, RdRp mechanism, RdRp structure, and other. We also observed a range of methodologies from in silico (i.e., computer modeling and simulations) to in vitro (i.e., laboratory experiments in cell cultures) to in vivo (i.e., experimental studies performed on mice or monkeys) to human clinical trials (from preliminary pilot studies to phase III trials). Accordingly, the question of whether the findings may be dependent on their study methods arose. For instance, whether compounds predicted to effectively inhibit viral replication in simulated computer models would in fact inhibit RNA synthesis in vitro or in vivo or whether the computer models may inaccurately predict inhibition based on including or excluding certain influential factors in the real world. 

Since the in silico studies often concluded that their findings should be further confirmed with in vitro, in vivo, and human clinical trial methods, the reviewers decided by consensus to focus the review on the primary themes that offer insights derived from multiple studies that used a variety of experimental methods. Consequently, a post hoc eligibility criterion was created requiring that studies had to be from a primary theme for which there were at least five studies that used in vitro or in vivo methods. Three reviewers (B.A.C., F.L.K., and A.S.) evaluated the full-text articles of the remaining records and categorized each article by the primary themes associated with the article’s research question, as well as the experimental methods used (i.e., in silico, in vitro, in vivo, or human clinical trial). This post hoc eligibility criterion was created with the intention of assessing whether the findings may be dependent on the experimental methods and/or whether conclusions about inhibitory behaviors may be consistent across studies. 

### 2.3. Data Charting and Synthesis

Two reviewers (B.A.C. and R.J.L.) jointly developed a data extraction (charting) form for the eligible studies to help create a descriptive summary of results that could address the scoping review objectives. Variables for which data were sought included author; publication year; country of origin; whether in silico, in vitro, in vivo, and/or human clinical trial methods were used; study aim; key methods or test type (e.g., molecular docking, SARS-CoV-2 inhibition assays, or human lung epithelial cell cultures); name(s) of the tested drug or investigational compound(s); outcome measures; key findings that related to the research questions; and which primary themes were addressed. Studies were grouped for syntheses based on shared experimental methods and/or key findings. Three reviewers (B.A.C., F.L.K., and A.S.) independently charted the data on Excel documents; they compared data charts and discussed any discrepancies. The final study selections were determined by consensus. 

## 3. Results

### 3.1. Overview of the Search Process

**Figure 1:**
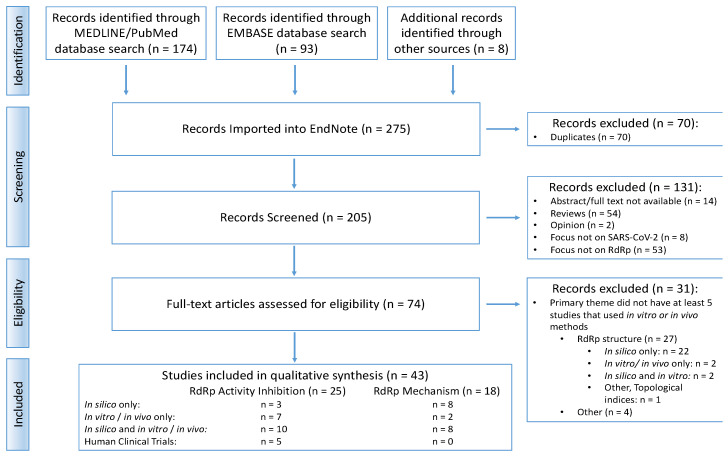
Flow chart of the study selection process.

Once the search strategies were employed in the MEDLINE/PubMed and EMBASE databases, 275 records were identified (Figure 1). After duplicates were removed, 205 titles and abstracts were screened for relevance, and the preliminary exclusion criteria were applied. The primary themes and experimental methods of the remaining 74 articles were then evaluated and categorized. Four primary themes were noted: investigation of RdRp activity inhibition, RdRp mechanism, RdRp structure, and other. Of these, two themes (RdRp activity inhibition and RdRp mechanism) met the post hoc eligibility criterion of having at least five studies that used in vitro or in vivo methods. 

Studies related to the other two primary themes (RdRp structure and other) did not provide sufficient evidence from in vitro or in vivo methods. Furthermore, 82% of studies that investigated RdRp structures were solely conducted in silico; only four studies utilized in vitro or in vivo methods, which was below the five-study minimum criterion. As such, studies related to these two primary themes were excluded from further review. 

Studies that focused on RdRp activity inhibition and RdRp mechanisms included evidence obtained through multiple experimental methods. Twenty-five articles explored the primary theme of RdRp activity inhibition through in silico simulations (n = 3), in vitro or in vivo experiments (n = 7), a combination of these methods (n = 10), or through human clinical trials (n = 5). Eighteen articles investigated the primary theme of RdRp mechanism through in silico simulations (n = 8), in vitro or in vivo experiments (n = 2), or a combination of these methods (n = 8). Thus, 43 articles met all the eligibility criteria and were included in the present scoping review synthesis. 

### 3.2. Summary of Findings

In the 43 articles included in the current scoping review (Table 1), 53 different drugs, compounds, or derivatives were tested in silico, in vitro, in vivo, or in human clinical trials to address the functional implications of RdRp and SARS-CoV-2. The authors were affiliated with institutions in 21 countries: Bangladesh, Belgium, Brazil, Canada, China, Egypt, France, Germany, Greece, India, Italy, Poland, the Republic of Korea, Russia, Saudi Arabia, Spain, Thailand, Ukraine, the United Kingdom, the United States of America, and Vietnam. 

The primary focus of each article was to either (1) identify existing drugs or compounds that could disrupt SARS-CoV-2 viral replication or (2) explore the underlying mechanisms of existing drugs or compounds that could inhibit SARS-CoV-2 RdRp enzymatic processes. 

#### 3.2.1. Adenosine-like Fungal Species

##### Cordycepin

Three in silico studies predicted that cordycepin, a natural adenosine analog found in fungal species that closely resembles normal nucleosides and nucleotides, can potentially bond with RdRp residues by resembling adenosine and can terminate viral replication early through ambiguous coding. In vitro experiments confirmed that cordycepin exhibits antiviral properties that are not only stronger than those of remdesivir but also effective against variants of concern [26,27,28].

1. Through molecular dynamics simulations and pharmacokinetics predictions, Bibi et al. (2022) found that cordycepin has the potential to be a strong and stable RdRp inhibitor through hydrophobic interactions and hydrogen bonding with residues in the RdRp active site [26].
2. Rabie (2022a) found that cordycepin has the ability to infiltrate SARS-CoV-2 genetic strands and stop the viral replication process by promoting ambiguous coding or early termination. The metabolic and structural similarities between cordycepin and nucleoside adenosine permit this incognito integration, as many enzymes cannot distinguish cordycepin triphosphate from ATP. Since the cordycepin molecule lacks a hydroxyl group, it disrupts SARS-CoV-2 RdRp activity, effectively terminating RNA synthesis and generating inactive, noninfectious material instead. Through in vitro bioactivity assays, Rabie found that cordycepin demonstrates stronger antiviral properties against SARS-CoV-2 than remdesivir. Long-lasting activities observed in vitro also validate the in silico findings that cordycepin exhibits high metabolic stability. Rabie suggested that cordycepin should be considered as a potential COVID-19 therapeutic due to its ability to target three main areas that contribute to SARS-CoV-2 infection: the spike protein, Mpro, and RdRp. Moreover, because cordycepin is nonspecific, in contrast to therapeutics that specifically target the spike protein, it can be effective against SARS-CoV-2 variants; this study showed inhibitory behaviors against variants of concern, including VOC-202012/01 [27].
3. Li et al. (2022) noted that cordycepin may inhibit SARS-CoV-2 replication, because it replaces the 3′-hydroxyl group with a hydrogen atom, and once it is incorporated into the new RNA strand (after mimicking a natural nucleotide), it prompts replication termination. Among the series of adenosine analogs tested, including the HIV inhibitor didanosine and the leukemia drug clofarabine, didanosine and cordycepin may be considered the best candidates to thwart nucleotide addition at the SARS-CoV-2 RdRp active site [28].

#### 3.2.2. Antibodies

##### Engineered Super-Antibody to Hepatitis C RdRp

4. Glab-ampai et al. (2022) evaluated the efficacy of an engineered super-antibody to hepatitis C RdRp against SARS-CoV-2 strains (Alpha, Beta, Delta, and Omicron), among other RNA viruses. Molecular docking and dynamic simulations revealed that this super-antibody can disrupt RdRp enzymatic activity through allosteric changes to the spatial conformation, thereby inhibiting viral replication. In vitro, cells infected with different SARS-CoV-2 variants were treated with the hepatitis C super-antibody, and viral RNA quantification showed a dose–response relationship; viral load reduction depends on the concentration of super-antibodies [29].

##### Poliovirus Vaccine That Induces Antibodies against Poliovirus RdRp

5. In a retrospective in vitro study, Comunale et al. (2021) examined whether serum samples from individuals recently vaccinated with the poliovirus vaccine could inhibit SARS-CoV-2 replication. Comparing sera from individuals who had not recently been vaccinated with those who had, enzyme-linked immunosorbent assays (ELISA) showed that antibody titers (including anti-RdRp) significantly increase with poliovirus vaccination. Using fluorescence-based RdRp assays, Comunale et al. also tested the ability of polio-immune sera to inhibit viral SARS-CoV-2 RNA synthesis and found that 76.5% of the tested samples could disrupt SARS-CoV-2 RdRp activity. Moreover, cytopathic effect (CPE)-based antiviral assays in Vero E6 cells demonstrated that polio-immune sera can inhibit SARS-CoV-2-induced CPE [30].

#### 3.2.3. Antifungal Antibiotics

##### Biosynthetic Gene Cluster of NPP B1

6. Park et al. (2022) explored the antiviral capabilities of a biosynthetic gene cluster of NPP B1, a compound similar to amphotericin B, which is an antifungal medication that is often used to treat cryptococcal meningitis in HIV-positive patients [31]. Amphotericin B has previously demonstrated antiviral properties against enveloped viruses, such as HIV, Japanese encephalitis, and rubella [32,33]; therefore, Park et al. tested NPP B1′s ability to inhibit viral SARS-CoV-2 RNA synthesis using a fluorescence-based RdRp assay in vitro. NPP B1 can exhibit more than 50% inhibition of SARS-CoV-2 RNA synthesis compared to a control (no antibiotics), but other antibiotics (kanamycin, aminoglycoside, beta-lactam, or ampicillin) may not exhibit any inhibitory behaviors [34].

#### 3.2.4. Antimalarials

##### Chloroquine and Derivatives (i.e., Hydroxychloroquine)

7. Through molecular docking and homology modeling, Nimgampalle et al. (2021) assessed the binding efficiencies of chloroquine and its derivatives to SARS-CoV-2 proteins. They found that chloroquine and hydroxychloroquine—antimalarial drugs that are also used to treat rheumatoid arthritis and lupus [35]— can bind to SARS-CoV-2 proteins like RdRp and have the potential to block viral replication by interfering with the catalytic active site. Moreover, derivatives such as CQN2H exhibited strong binding to RNA polymerase, as well as hydrogen bonding to RNA polymerase residues THR319 and THR394, suggesting that derivatives may also be potential SARS-CoV-2 inhibitors [36].
8. Maisonnasse et al. (2020) tested hydroxychloroquine’s antiviral properties against SARS-CoV-2 in Vero E6 cell cultures and in infected macaques. None of the analyzed tissues displayed significant inhibitory properties against SARS-CoV-2 either in vitro or in vivo. Hydroxychloroquine was tested as a prophylactic drug and as a possible treatment, both alone and in combination with azithromycin, an antibiotic used to fight bacterial infections. In vitro and in vivo results suggested that these therapeutic strategies may not be clinically beneficial, as the drugs do not significantly reduce viral loads or accelerate time to viral clearance [37].
9. Yao et al. (2020) assessed the antiviral and prophylactic properties of chloroquine and hydroxychloroquine against SARS-CoV-2 in Vero E6 cells in vitro. Physiologically based pharmacokinetic (PBPK) models were also used to analyze potential dosing regimens. Yao et al. found that hydroxychloroquine inhibits SARS-CoV-2 in vitro and displays stronger inhibitory behaviors than chloroquine against SARS-CoV-2. The most effective simulated dosing regimen was noted as a loading dose of 400 mg twice per day, followed by 4 days of 200 mg given twice daily. Yao et al. suggested that hydroxychloroquine’s prophylactic activities, antiviral potency against SARS-CoV-2, and safety profile make it a promising therapeutic candidate to combat cytokine storms in patients who are critically ill [38].
10. Gautret et al. (2020) studied the effect of hydroxychloroquine on respiratory viral loads in a non-randomized clinical trial with 36 patients. Validating prior research that chloroquine and its derivative hydroxychloroquine can inhibit SARS-CoV-2 viral replication in vitro, 70% of the patients who received hydroxychloroquine recovered from COVID-19 within six days compared to only 12.5% of patients in the untreated group. As such, researchers concluded hydroxychloroquine can successfully reduce SARS-CoV-2 viral loads, and the effects may be further bolstered by azithromycin [39].

While both studies that used in silico methods were consistent in predicting that chloroquine and hydroxychloroquine could bind at the SARS-CoV-2 RdRp active site, suggesting the potential to disrupt SARS-CoV-2 viral replication, the findings from the in vitro studies diverged [36]. Yao et al. (2020) found that hydroxychloroquine could be effective as a prophylaxis and/or treatment against SARS-CoV-2 in vitro, with dosing regimens predicted by PBPK models [38]. However, Maisonnasse et al. (2020) did not find any significant inhibitory properties of hydroxychloroquine as a prophylaxis or treatment against COVID-19, in vitro, or in infected macaques [37].

#### 3.2.5. Antioxidants

##### C60 Fullerene

Hurmach et al. confirmed through two studies (2021a and 2021b) that C60 fullerene, a compound used as an antioxidant [40,41], can inhibit SARS-CoV-2 in silico and in vitro by blocking cell entry and disrupting the formation of the RdRp-nsp8 complex through stacking and steric interactions [42,43].

11. Through molecular docking and simulations, Hurmach et al. (2021a) found that C60 fullerene, a compound often used as an antioxidant, is capable of inhibiting RdRp activity in silico. Dynamic simulations suggested two possible mechanisms of RdRp inhibition: (1) C60 fullerene blocks the RNA synthesis channel with its bulky carbon structure, thus thwarting normal replication processes, and (2) through stacking and steric interactions, C60 fullerene binds to two pockets that are critical to forming the RdRp-nsp8 complex, which assists in RNA synthesis. As SARS-CoV-2 RdRp (nsp12) cannot synthesize RNA on its own, it needs to form a complex with other nonstructural proteins, such as nsp7 and nsp8. Failure to create this complex results in an inability to replicate viral RNA [42].
12. Utilizing molecular dynamics and microscopic, spectroscopic, and in vitro methods, Hurmach et al. (2021b) evaluated the efficacy of nanostructure C60 fullerene against coronaviruses. In silico methods showed that the molecular structure of C60 fullerene is comprised of 60 carbon atoms that form a bulky, spherical shape. As such, C60 fullerene can block the coronavirus from entering the cell. It can also fill the RdRp binding pocket and effectively prevent RNA synthesis through stacking and steric interactions. Hurmach et al. found that C60 fullerene has the ability to decrease titers of infectious activity, can form stable complexes with SARS-CoV-2 RdRp through direct interactions, and can inhibit SARS-CoV-2 RdRp functionality [43].

##### Flavonoids (i.e., Quercetin, Theaflavin, and Luteolin)

Two studies explored the potential of quercetin, a plant flavonol with anti-inflammatory properties [44], as an agent against SARS-CoV-2. Both in vitro experiments suggested that viral replication may be reduced in a dose-dependent manner. In silico modeling suggested efficacy due to stable bond formations at RdRp binding pockets [45,46].

13. Goc et al. (2022) evaluated the inhibition of RdRp activity from a mixture of natural compounds, consisting of vitamin C, N-acetylcysteine, resveratrol, theaflavin, curcumin, quercetin, naringenin, baicalin, and broccoli extract, in the hopes that a mixture of compounds could offer a multilayered protection effect against SARS-CoV-2. Using fluorescence-based RNA polymerase assay kits, Goc et al. found that the natural compound mixture is effective in inhibiting both the original and Omicron SARS-CoV-2 strains, though the strongest contributors to such inhibition are quercetin, theaflavin, and curcumin. Cytotoxicity and cell viability were tested in human alveolar epithelial cells, showing that quercetin and theaflavin are the most potent components targeting the RdRp complex. While the degree of inhibition was dose-dependent, an ideal dose of 0.1 mg/mL resulted in nearly 100% inhibition [45].
14. Munafò et al. (2022) explored whether quercetin and luteolin display antiviral properties against SARS-CoV-2 RdRp both in vitro and in silico. The anti-inflammatory properties of flavonoids suggest that SARS-CoV-2 inhibition may be demonstrated against several viral proteins, such as the Mpro and spike proteins. However, Munafò et al. observed that inhibitory behaviors are stronger against the RdRp protein than the Mpro and spike proteins at varying concentrations (total inhibition at 100 µM; 80% inhibition at 25 µM). Molecular dynamics simulations further validated the in vitro findings, suggesting that both quercetin and luteolin can form stable bonds at RdRp binding pockets [46].

##### Taroxaz-104

15. The Taroxaz-104 molecule is an antioxidant compound that is highly effective in transporting and mediating zinc uptake, which can play a direct role in inhibiting viral RdRp activity. Rabie (2021) noted that in silico predictions of Taroxaz-104 were highly effective against SARS-CoV-2 by interacting with several amino acids in the RdRp active site. In vitro, CPE-based antiviral bioassays in Vero E6 cells confirmed that Taroxaz-104 can successfully inhibit SARS-CoV-2 replication and transcription more than the reference compound, GS-443902, the active metabolite of RdRp inhibitor remdesivir. Furthermore, Taroxaz-104 demonstrates 43 times the potency of GS-443902 against the VOC-202012/01 SARS-CoV-2 variant. The ability of Taroxaz-104 to hinder SARS-CoV-2 RNA replication processes and inhibit CPEs indicates the powerful potential to repurpose this drug against SARS-CoV-2 variants. As fundamental mutations to the SARS-CoV-2 spike protein have been observed across variants, Rabie recommended utilizing drugs that inhibit key SARS-CoV-2 proteins that are not located on the viral surface, such as RdRp [47].

#### 3.2.6. Antiparasitics

##### Suramin

16. Yin et al. (2021) explored whether and how suramin, a drug used to treat African sleeping sickness and river blindness [48], could inhibit SARS-CoV-2 replication through in silico and in vitro methods. Structural modeling demonstrated RdRp–suramin binding at two sites, both of which are needed for viral replication. At one site, where RNA template strands would bind, the RdRp–suramin complex blocks natural substrates from entering the active site, directly ceasing viral replication. Then, near the RdRp catalytic site, the RdRp–suramin complex conflicts with the RNA primer strand and indirectly prevents RdRp enzymatic processes from occurring. In vitro experiments in Vero E6 cells confirmed that suramin can inhibit SARS-CoV-2 viral replication [49].

#### 3.2.7. Antivirals

##### BMS-986094 (Developed for Hepatitis C) [50]

17. Chemical structure modeling by Jimenez-Guardeño et al. (2022) suggested that BMS-986094, an RdRp inhibitor initially developed for the hepatitis C virus [51,52], and different forms of vitamin B12 may be effective against SARS-CoV-2. While quadratic unbounded binary optimization (QUBO) and Tanimoto models predicted inhibitory behaviors in silico, SARS-CoV-2 RNA polymerase and cytotoxicity assays in vitro also suggested that each tested compound could effectively disrupt the viral replication of four strains: SARS-CoV-2 Strain England 2 (England 02/2020/407073), B.1.1.7 (Alpha), B.1.351 (Beta), and B.1.617.2 (Delta) [53].

##### Remdesivir (Developed for the Ebola Virus) [54]

18. Pirzada et al. (2021) examined U.S. Food and Drug Administration-approved RdRp inhibitors as potential therapeutics for SARS-CoV-2. In silico methods suggested that remdesivir, a drug developed to treat Ebola [55,56], as well as two hepatitis C drugs, ledipasvir and paritaprevir [57,58], may exhibit strong interactions with SARS-CoV-2 RdRp. Subsequently, the antiviral properties of all three drugs were tested in Vero E6 cell cultures, and the findings indicated that, compared to ledipasvir and paritaprevir, remdesivir is the most potent SARS-CoV-2 RdRp inhibitor [59].
19. Vangeel et al. (2022) found that remdesivir, molnupiravir, and nirmatrelvir (the antiviral active ingredient in Paxlovid), three SARS-CoV-2 antivirals that target either the RdRp or Mpro proteins, demonstrate similar efficacy and potency in vitro across five of the main SARS-CoV-2 strains (Alpha, Beta, Gamma, Delta, and Omicron). Some of the structural characteristics that distinguish the original (Alpha) SARS-CoV-2 strain from variants of concern are associated with amino acid changes that are far from the RdRp and Mpro active sites. Thus, RdRp and Mpro inhibitor drugs can remain effective regardless of which SARS-CoV-2 strain one may be exposed to, because the target proteins are highly conserved. Vangeel et al. suggested that more antivirals that target proteins other than the commonly addressed spike protein, such as RdRp or Mpro, should be strongly considered, as the drugs are more likely to remain effective amidst new variants [60].
20. Through molecular docking methods, Choudhury et al. (2020) investigated which known RdRp inhibitors of other viruses may be effective in disrupting SARS-CoV-2 replication in silico. Ligand–receptor interaction docking scores predicted which compounds could form stable complexes and/or have high affinities for the RdRp active site and thus may interfere with the usual enzymatic activity processes. Remdesivir was noted as the strongest inhibitor, having the ability to compete with natural substrates and become incorporated into RNA chains, thereby terminating replication. Chlorhexidine, a drug commonly used for dental plaques, gingivitis, and tonsillitis, also demonstrated strong affinities for the RdRp active site, though the mechanism to inhibit RdRp was not by terminating replication via substrate competition but rather by actively blocking the RdRp cavity and preventing further replication. Additionally, Choudhury et al. predicted five other nucleoside analogs that are similar to remdesivir that could be effective in inhibiting SARS-CoV-2 RdRp activity: sofosbuvir, ribavirin, penciclovir, ganciclovir, and favipiravir [61].
21. Zhang and Zhou (2020) found that remdesivir may act as an effective inhibitor against SARS-CoV-2 in the same manner that it can stop reproduction of the Ebola virus. Once remdesivir is metabolized in the body (hydrolyzed and phosphorylated), it can act as a substitute for ATP, given its similar structure to adenosine, and thus may serve as a binding substrate that can obstruct the “grip” of SARS-CoV-2 RdRp, prompting the cessation of RdRp activity and RNA replication [62].
22. To better understand the interactions between remdesivir and SARS-CoV-2 RdRp residues that may contribute to viral replication inhibition, Koulgi et al. (2020) compared RdRp conformations in the apo form (unbound state) with the remdesivir-bound form in silico. Ensemble docking and molecular dynamics simulations revealed opposite movements: in the apo form, the template entry site is exposed to give natural nucleotides access; in the remdesivir-bound form, hydrogen bonding and residue contacts obstruct natural nucleotides from accessing the cavity, as the template entry site is filled by remdesivir, prompting viral replication termination [63].
23. Nguyen et al. (2020) were interested in how remdesivir binds to two key SARS-CoV-2 proteins, RdRp and Mpro, in silico. Molecular docking predicted that Mpro would have a stronger bond with remdesivir than RdRp would, but molecular dynamics simulations showed the converse to be true. Nguyen et al. suggested that this discrepancy may be due to molecular docking methods incorrectly estimating the binding energies compared to more advanced methods like molecular dynamics simulations. Since remdesivir’s binding site is close to the RdRp active site, remdesivir can easily form hydrogen bonds, as well as approach and obstruct the open RdRp cavity. Nguyen et al. also found that remdesivir binds to each key protein in a different manner: electrostatic interactions stabilize the RdRp–remdesivir complex, while van der Waals interactions are responsible for stabilizing Mpro binding [64].
24. Aranda et al. (2022) sought to characterize the mechanism of reaction in SARS-CoV-2 RdRp with natural triphosphates and remdesivir triphosphates in silico. Contrary to the popular hypothesis that remdesivir inhibits SARS-CoV-2 viral replication by competing with ATP, Aranda et al. suggested that a covalent bond with RdRp at position i+4 may be responsible for the observed delayed inhibition instead of the widely accepted steric clashing. Moreover, the remdesivir triphosphate incorporation rate inside RdRp can be slower than that of natural triphosphate (ATP). Through equilibrium trajectories, Aranda et al. noted that remdesivir triphosphate is the same size and shape as adenosine, exhibits strong interactions with uridine triphosphate (UTP), and is well suited for the RdRp active site; the simulations displayed remdesivir triphosphate incorporation into nascent RNA in front of UTP, contradicting findings that the substrate blocks RdRp. While the in silico findings suggested new mechanisms to explain inhibitory behaviors, the data nonetheless validated that remdesivir can be an effective drug against SARS-CoV-2 by targeting RdRp [65].
25. Tchesnokov et al. (2020) studied remdesivir’s role in SARS-CoV-2 viral replication inhibition in vitro. They found that remdesivir is capable of disrupting SARS-CoV-2 RdRp enzymatic processes once remdesivir triphosphate is incorporated into the RNA primer strand, as it is later used as a template for RNA synthesis. Once the remdesivir residue is in the template strand, nucleotide incorporation is notably diminished. Moreover, delayed chain termination may be observed with low nucleoside triphosphate (NTP) concentrations; when NTP is increased, the translocation equilibrium changes, which consequently unveils nucleotide binding sites. As such, increasing UTP and ATP, or the UTP, ATP, and cytidine triphosphate (CTP) concentrations, erodes the inhibitory properties, enhances read-through, and enables full-length RNA product development [66].
26. Through sequence analyses, Stevens et al. (2022) explored nonsynonymous mutations (nucleotide sequence changes) in SARS-CoV-2 RdRp and sought to understand the possible mechanisms behind the resistance to remdesivir. Thus far, SARS-CoV-2 variants in the natural environment have not exhibited resistance to remdesivir, but there is a possibility that remdesivir resistance mutations could arise in minority variants. Stevens et al. found that the mutations V792I and S759A in SARS-CoV-2 RdRp could exhibit resistance to remdesivir. The V792I mutant may minimize the template-dependent inhibition of viral replication by lowering UTP concentrations. The S759A mutant is significantly less likely to use remdesivir triphosphate as a substrate compared to ATP (10.5-fold decrease), thereby diminishing the utility of the RdRp inhibitor. However, the S759A mutation was not found among the Delta and Omicron variant sequences, and other substitutions were identified at low frequencies, suggesting that contemporary variants do not show resistance to the RdRp inhibitor remdesivir [67].

While the majority of studies that have examined remdesivir confirmed that it is a potent SARS-CoV-2 RdRp inhibitor [62,63,64,65,66,67], several studies have found that other drugs or compounds may be more effective in silico and/or in vitro: suramin [49], Taroxaz-104 [47], cordycepin [27], didanosine [68], gossypol [69], and sofosbuvir [70].

Additionally, the mechanism by which remdesivir may inhibit SARS-CoV-2 was not consistent across studies. While some in silico findings suggested competition with natural substrates [61,62], other in silico and in vitro studies proposed that inhibition is prompted by hydrogen bonding and blocking the RdRp cavity [63,64] or covalent bonding [65].

##### Molnupiravir (Developed for the Influenza Virus) [71] and Derivatives (i.e., 4′-Fluorouridine and N4-Hydroxycytidine)

Four in vitro studies confirmed that molnupiravir, a N4-hydroxycytidine (NHC) prodrug originally developed to treat influenza, can inhibit SARS-CoV-2 RdRp enzymatic activities. Once incorporated into the template strand, it can create dysfunctional genomes that disrupt normal viral replication processes [72], thereby reducing viral loads and infectious titers in vitro and in vivo [73,74], as well as across variants [60,74].

27. As mentioned above, Vangeel et al. (2022) found that molnupiravir has efficacy and potency comparable to remdesivir and nirmatrelvir (found in Paxlovid) in vitro across the SARS-CoV-2 strains (Alpha, Beta, Gamma, Delta, and Omicron). As the amino acid changes that differentiate strains are far enough away from the RdRp active site, RdRp inhibitors can continue to successfully interact with the RdRp protein and inhibit viral replication [60].
28. Sourimant et al. (2022) analyzed the antiviral properties of 4′-fluorouridine (4′-FlU; EIDD-2749), a ribonucleoside analog and derivative of molnupiravir, against several RNA viruses, including respiratory syncytial virus (RSV) and SARS-CoV-2. In vitro, Sourimant et al. found that 4′-FIU incorporation can prompt an antiviral effect of RNA polymerase stalling. In vivo, viral titers in nasal turbinate tissue revealed that a daily dose of 20 mg/kg of 4′-FIU can effectively reduce the viral load and inhibit SARS-CoV-2 replication in infected ferrets across the Alpha, Gamma, and Delta variants [74].
29. N4-hydroxycytidine (NHC; EIDD-1931), a ribonucleoside analog, has antiviral properties against several RNA viruses, such as influenzas A and B, Ebola, and Venezuelan equine encephalitis virus [75,76]. Sheahan et al. (2020) explored whether NHC is capable of inhibiting SARS-CoV-2 in vitro and in vivo. Utilizing human airway epithelial cell cultures, they found that NHC reduces infectious titers and viral SARS-CoV-2 RNA in a dose-dependent manner. Additionally, a prophylactic dose escalation in vivo study in mice suggested that an orally bioavailable NHC prodrug (EIDD-2801, also known as molnupiravir) notably decreases lung hemorrhaging, SARS-CoV lung titers, and viral loads. Surrogate markers for pulmonary obstruction can also be enhanced, though clinical improvement may be dependent on when NHC is administered post-infection [73].
30. Gordon et al. (2021) evaluated the mechanism of how molnupiravir (NHC 5′-triphosphate (NHC-TP)) promotes antiviral effects against SARS-CoV-2 in vitro. They observed that NHC-TP competes with CTP for incorporation (a C-analog), but when NHC is in the monophosphate form (NHC-MP) and embedded in the template strand, it can compete with CTP and UTP, thus favoring the incorporation of guanosine triphosphate (GTP) and ATP. Then, when GTP is incorporated opposite NHC-MP, subsequent nucleotides are unable to be incorporated into the strand (i.e., viral inhibition commences). When NHC-MP competes with UTP and favors the incorporation of ATP, NHC:A base pairs lead to transition mutations in G to A and in C to U. These mutation frequencies ultimately produce dysfunctional genomes that then disrupt normal replication processes. Thus, Gordon et al. showed that molnupiravir is responsible for mutagenesis when embedded into the template strand. Conversely, when NHC-MP is embedded in the RNA primer strand, it does not facilitate RNA synthesis inhibition [72].
31. Khoo et al. (2023) evaluated the safety and efficacy of molnupiravir against various SARS-CoV-2 strains in a phase II randomized, double blinded placebo-controlled clinical trial. Those who were treated with molnupiravir recovered from COVID-19 significantly faster than those who received the placebo (8 days versus 11 days, respectively). Additionally, no participants in the treatment group were hospitalized compared to four participants in the placebo group. While preliminary evidence indicates molnupiravir is well tolerated and may be effective against COVID-19, the predefined threshold was not reached, prompting Khoo et al. to conclude that the evidence may not be conclusive [77].

##### Nucleoside Analogs (i.e., Didanosine, Fludarabine, Vidarabine, and Favipiravir)

Two studies involving didanosine confirmed that the HIV inhibitor can act as a chain terminator for SARS-CoV-2 RNA synthesis and can display stronger potency in vitro against variants of concern compared to the commonly used COVID-19 treatment remdesivir [28,68]. Additionally, three human clinical trials evaluated the efficacy of favipiravir in mild-to-moderate COVID-19 cases.

32. To understand how 2′- and 3′-ribose modifications of adenosine analogs may inhibit SARS-CoV-2 replication, Li et al. (2022) conducted molecular dynamics simulations of nucleotide analogs that target RdRp, including drugs used for leukemia (clofarabine and fludarabine) [78,79], HIV inhibitors (didanosine and 2′,3′-didehydro-2′,3′-dideoxyadenosine) [80,81], and inhibitors used against herpes simplex virus and varicella-zoster virus (vidarabine) [82]. They found that a 2′-ribose modification affects not only the binding stability but also the substrate incorporation efficiency into the new RNA strand. Additionally, 3′-ribose modifications, such as a 3′-hydroxyl group removal, as seen in didanosine and 2′,3′-didehydro-2’,3’-dideoxyadenosine, can also enable inhibition. Didanosine and cordycepin, natural adenosine analogs found in fungal species, were noted as the strongest agents to disrupt nucleotide addition at the SARS-CoV-2 RdRp active site [28].
33. Rabie (2022b) investigated the effectiveness of didanosine against SARS-CoV-2 RdRp both in vitro and in silico. Molecular docking and computational interpretations revealed that didanosine interacts with a fundamental pocket of the RdRp active site and is incorporated into new viral strands instead of natural substrates (ATP and GTP), thereby creating defective viral particles and prompting the termination of SARS-CoV-2 RNA synthesis. The molecular makeup of didanosine aids in these processes, as it lacks a 3′-hydroxyl group, and such a deficiency is often associated with premature RNA synthesis termination. In silico data confirmed what Rabie observed in vitro through antiviral assays in Vero E6 cells against the VOC-202012/01 SARS-CoV-2 variant strain: didanosine not only suppressed viral replication but also proved to be more potent (by 5.0-6.8 times) than remdesivir against the SARS-CoV-2 variant [68].
34. Sirijatuphat et al. (2022) investigated the efficacy of favipiravir, a nucleoside-based antiviral drug that is commonly used to treat influenza, among 96 patients that had COVID-19, had not received COVID-19 vaccinations, and did not have pneumonia at the onset of the study. The potential effects on viral clearance, their clinical condition, and the risk of developing pneumonia were evaluated. Validating prior in vitro analyses of inhibitory activity, patients who received favipiravir showed clinical improvements in two days compared to those in the control arm at fourteen days. Sirijatuphat et al. asserted favipiravir can expedite recovery in mild COVID-19 cases and is safe for short-term use [83].
35. Udwadia et al. (2020) conducted a phase III clinical trial testing the efficacy, safety, and clinical benefits of favipiravir among 150 patients. The median time of SARS-CoV-2 viral shedding was five days for those who received favipiravir compared to seven days for those in the control arm. While this difference was not statistically significant, the results were clinically meaningful for the secondary endpoint, the median time to a clinical cure, with three days for patients who received favipiravir and five days for those who did not. Researchers suggest favipiravir is not only safe and well-tolerated but may also be beneficial in mitigating the effects of mild-to-moderate COVID-19 [84].
36. Shinkai et al. (2021) assessed the efficacy and safety of favipiravir against COVID-19 among patients with moderate pneumonia in a randomized placebo-controlled Phase III trial. Researchers evaluated the median time of recovery, as measured by an improvement in temperature, oxygen saturation levels (SpO2), findings on chest imaging, and testing negative for SARS-CoV-2. Patients who were treated with favipiravir recovered significantly faster than those who received the placebo (11.9 days versus 14.7 days, respectively), suggesting favipiravir may be considered clinically effective for COVID-19 pneumonia patients [85].

##### Sofosbuvir (Developed for Hepatitis C) [86]

Four studies confirmed that sofosbuvir can inhibit SARS-CoV-2 viral replication in silico and/or in vitro [61,70,87,88].

37. As mentioned above, Choudhury et al. (2020) found that sofosbuvir, a drug commonly used to treat hepatitis C [89], is effective in disrupting SARS-CoV-2 replication processes in silico [61].
38. Yuan et al. (2021) analyzed how five different 2′-modified nucleotides, including sofosbuvir and gemcitabine, a chemotherapy drug [90], may affect the polymerization process and/or inhibitory behaviors of SARS-CoV-2 RdRp. Considering 2′-O-methyl UTP, 2′-C-methyl CTP, gemcitabine, ara-UTP, and sofosbuvir, Yuan et al. found that the size and shape of 2′-methyl substitutions in 2′-C-methyl CTP and sofosbuvir greatly disrupt the polymerization processes once they are incorporated into the active site, so much so that the polymerase chain is instantly terminated from steric hindrance. On a lesser scale, 2′-O-methyl UTP may serve as a partial chain terminator, because molecular binding may be hindered upon incorporation. Furthermore, smaller 2′-substitutions, such as those in ara-UTP and gemcitabine, do not effectively inhibit viral replication [88].
39. Similarities between SARS-CoV-2 RdRp and hepatitis C enzymes NS5A (protease) and NS5B (RdRp) led Sacramento et al. (2021) to investigate whether drugs targeting these enzymes, such as daclatasvir and sofosbuvir [91,92,93], could also inhibit SARS-CoV-2 viral replication. Molecular docking suggested that both drugs can interact with SARS-CoV-2 RNA residues through hydrogen bonding and indicated that steric interactions exist for both compounds. In vitro assays in Vero E6 cells, human lung epithelial cells, and human hepatoma lineage cells demonstrated that daclatasvir can inhibit SARS-CoV-2 RNA synthesis and that sofosbuvir has antiviral capabilities, though the effects may be stronger when combined with daclatasvir than when administered alone. The inhibitory mechanisms differ between the two drugs: sofosbuvir acts as a chain terminator, and daclatasvir targets folds in the RNA secondary structures, inciting destabilization and disrupting polymerase reactions. Sacramento et al. suggested further investigation into dosing for SARS-CoV-2 treatment, as effective doses may be higher than those currently prescribed to treat hepatitis C [87].
40. SARS-CoV-2 has an exonuclease-based proofreader (nonstructural protein 14, nsp-14) that detects and removes incorrect nucleotides that have been incorporated into new viral strands. Many RdRp inhibitor drugs that exhibit antiviral properties against SARS-CoV-2 rely on the ability to mimic natural substrates, such as ATP, in order to become incorporated into viral RNA and prompt replication termination; therefore, their drug efficacy may be affected by nsp-14′s proofreading abilities. Jockusch et al. (2020) examined SARS-CoV-2 RNA that was terminated by either sofosbuvir, a hepatitis C inhibitor, or remdesivir, a therapeutic for Ebola, and compared which may be more resistant to nsp-14 and avoid removal so RNA synthesis could cease. Considering the active forms of each drug, Jockusch et al. found that sofosbuvir triphosphate is removed less frequently than remdesivir triphosphate, suggesting that sofosbuvir may be a promising therapeutic against SARS-CoV-2, because it is more resistant to nsp-14 than remdesivir [70].

##### Uracil Derivatives (Used for HIV and Hepatitis C) [94,95]

41. As non-nucleoside uracil derivatives have previously been effective antiviral agents against HIV, hepatitis C, and varicella zoster virus [96], Siniavin et al. (2022) investigated whether N1,N3-disubstituted derivatives of uracil could be utilized against SARS-CoV-2. In silico, compound 876 displayed the best score to predict the disruption of RdRp functionality (through placement in the hydrophobic pocket of the RNA cleft). In vitro, Siniavin et al. found that compound 876 can inhibit SARS-CoV-2 RdRp at varying concentrations (100% inhibition at 100 µM and 40% inhibition at 50 µM), and compounds 871, 874, and 1007 could decrease viral replication of the SARS-CoV-2 variants of concern (Beta, Delta, and Omicron) [97].

##### CRISPR Genome Editing

42. Abbott et al. (2020) proposed an alternative method to targeting SARS-CoV-2 to the currently available vaccines that aim to prime and help the immune system recognize proteins like the SARS-CoV-2 spike protein [98]. Instead, they suggested using prophylactic antiviral CRISPR in human cells (PAC-MAN) to edit the protein sequences of SARS-CoV-2 RNA so that intracellular viral genome templates that are needed for viral replication may degrade prophylactically. While in silico models identified specific, highly conserved SARS-CoV-2 genomic regions for the Cas13d enzyme to target, in vitro human lung epithelial cell cultures confirmed that PAC-MAN could inhibit viral gene expression across potential variants and mutations that may arise naturally through antigenic drift. Abbott et al. also employed this strategy against the influenza A virus and found that, by targeting the highly conserved RdRp genes, one could inhibit viral production and prompt viral genome deterioration, thereby reducing the viral load in human lung epithelial cells in vitro [99].

#### 3.2.8. Vitamins, Natural Compounds, and Extracts

##### Vitamin B12

In silico and in vitro studies examining vitamin B12 suggested it not only binds to the RdRp active site in place of natural triphosphate but also effectively inhibits the viral replication of SARS-CoV-2 variants, including the Alpha, Beta, Delta, and England 2 strains [53,100].

43. Narayanan and Nair (2020) utilized homology modeling and molecular dynamics simulations to identify vitamin B12 as a potential SARS-CoV-2 RdRp inhibitor. Since the binding site for vitamin B12 overlaps with that of NTPs, simulations predicted that vitamin B12 could bind to the SARS-CoV-2 RdRp active site in place of NTPs, therefore inhibiting polymerization processes. An analysis of the binding energies confirmed that vitamin B12 can bind to the RdRp active site with significant affinity [100].
44. As mentioned above, Jimenez-Guardeño et al. (2022) found that different forms of vitamin B12 can inhibit SARS-CoV-2 viral replication in silico and in vitro across variant strains, including SARS-CoV-2 Strain England 2 (England 02/2020/407073), B.1.1.7 (Alpha), B.1.351 (Beta), and B.1.617.2 (Delta) [53].

##### Gossypol (Used in Cancer Therapies) [101,102,103,104]

Gossypol, a natural phenol found in the cotton plant and unrefined cottonseed oil, has been used in China to treat gynecological diseases and is currently being tested to treat certain types of lung cancer [105,106]. Using computational screening, RNA extension assays, and gel-based RdRp assays, Wang et al. (2022) found the phenolic aldehyde compound gossypol to be a potent inhibitor against SARS-CoV-2, even when compared to remdesivir triphosphate. Gossypol not only effectively inhibited SARS-CoV-2 in human airway epithelial cell cultures but also did so in mouse infection models in vivo. Gossypol notably decreases SARS-CoV-2 viral loads and infectious titers in nasal turbinates and lungs. Cryo-electron microscopy indicated that, together, two gossypol molecules could crowd the SARS-CoV-2 RdRp active site, diminish the size of the central cavity opening, and obstruct the critical RdRp–RNA complex from developing. Additionally, inhibition assays in Vero E6 cells revealed that gossypol is also effective against SARS-CoV-2 variants of concern, such as Delta and Omicron [69].

##### Hypericin (Used in Cancer Therapies) [107]

45. Matos et al. (2021) utilized structure-based virtual screening to identify potential therapeutics against SARS-CoV-2 that target proteins critical to the viral replication process, such as RdRp. They noted six compounds with high affinities for the SARS-CoV-2 RdRp active site, most notably hypericin, which is a naturally occurring substance found in St. John’s wort that has previously been used as an antiviral agent and in cancer therapies [108]. In a dose–response in vitro experiment, Matos et al. demonstrated that, at the highest tested concentrations, hypericin can reduce viral RNA synthesis up to 96%. Moreover, LDH cytotoxicity assays were conducted in cultured cells and showed no significant plasma membrane damage, further confirming that hypericin may be a promising therapeutic agent against COVID-19 [109].

##### Corilagin (Used in Cancer Therapies) [110]

46. Li et al. (2021) examined corilagin, a non-nucleoside RdRp inhibitor that is found in medicinal plants and has been used to diminish cancerous cell growth [111], and its ability to inhibit SARS-CoV-2 polymerization both in silico and in vitro. Molecular modeling predicted that corilagin would inhibit RdRp activity by docking at the palm subdomain of RdRp, thereby blocking NTP from entering the SARS-CoV-2 active site. In both cell-free and cell-based assays, corilagin can disrupt SARS-CoV-2 viral replication. Moreover, corilagin and remdesivir demonstrate dose–response relationships, and when both are used in treatments, there is an additive effect in inhibiting RdRp [112].

Overall, in silico, in vitro, in vivo, and human clinical trial studies alike have predicted and experimentally tested compounds that could inhibit SARS-CoV-2 replication by targeting the RdRp protein. These existing drugs and compounds were consistently effective in inhibiting RdRp activity processes, with the exceptions of chloroquine and hydroxychloroquine.

**Figure 2:**
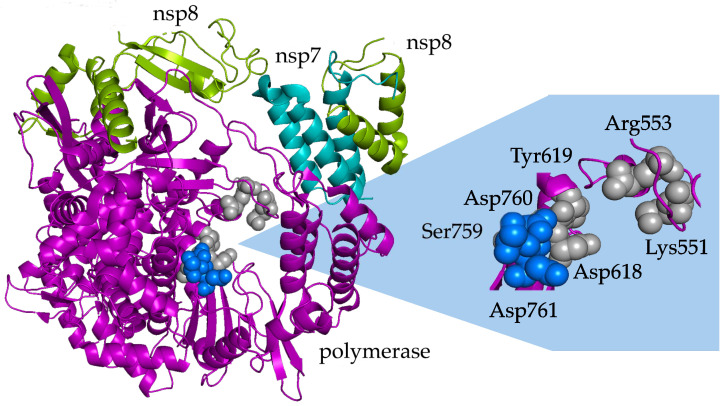
Key amino acid residues of the SARS-CoV-2 RNA-dependent RNA polymerase (RdRp) nsp12 complex with cofactors nsp7 and nsp8 interact with bioactive compound inhibitors targeting RdRp. Catalytic residues (highlighted in blue: Asp760, Asp761, and Ser759) and other active site residues (Asp618 and Tyr619) have the strongest contact with inhibitors via hydrophobic interactions and hydrogen bonding. Electrostatic and steric interactions are seen with amino acid residues positioned near the catalytic center of functional motif C (Arg553 and Lys551). When inhibitors interact with the residues of conserved motifs, the protein declines in functionality, as normal replication processes are disrupted.

Additionally, in silico studies have proposed possible mechanisms to explain these behaviors on the molecular level through simulations of steric hindrance, competition with natural substrates, formations of hydrogen or covalent bonding, and whether or not RdRp active sites may be blocked in the simulation. In vitro studies have further explored these mechanisms by examining RNA synthesis via primer or template strand incorporation; resistance to proofreading proteins; the inhibition potency; and reduction in the viral load, infectious titers, or enzymatic activities. The underlying mechanisms of RdRp inhibitory behaviors for three drugs—sofosbuvir, didanosine, and remdesivir—could be compared across studies. In silico and in vitro methods have validated that sofosbuvir acts as a chain terminator due to steric interactions at the RdRp active site, and didanosine terminates viral replication chains by competing with natural substrates and becoming incorporated into new RNA strands. However, discrepancies were found in understanding the mechanisms by which remdesivir may exhibit antiviral properties against SARS-CoV-2; some studies have suggested natural substrate competition, whereas others have proposed that hydrogen bonding or covalent bonding may be responsible for viral inhibition. Across the studies, several amino acid residues were continually credited for interacting with bioactive compound inhibitors, consequently contributing to loss in functionality of the RdRp protein. Figure 2 highlights these key amino acid residues, which are all located in or near the catalytic center of functional motif C.

## 4. Discussion

The purpose of this scoping review was to define the scope of the current literature on RdRp in the context of the COVID-19 pandemic, with specific interest in functional implications for future therapeutic targets. Our motivation included awareness that viral core proteins, such as RdRp, may provide more durable targets than surface proteins, which are known to change at a more rapid rate [113]. We found studies that used both simulated and biologically based methods to predict and/or demonstrate interactions with SARS-CoV-2 RdRp and disruptions of viral replication processes and whether the findings may vary by study methods. Twenty-five studies identified potential SARS-CoV-2 RdRp inhibitors, and eighteen studies explored possible mechanisms to explain the inhibitory behaviors through in silico, in vitro, in vivo, and clinical trial methods.

Simulated models predicted activities that led to the termination of SARS-CoV-2 synthesis via binding energies, interactions with RdRp residues, indications of stacking interactions, steric hindrance, competition with natural substrates, the formation of hydrogen or covalent bonding, and RdRp cavity accessibility.

In vitro studies assessed RdRp functionality via reduction in the viral load or infectious titers, incorporation into the RNA primer or template strands, the synthesis of new strands through emitted fluorescence intensity, the generation of ineffective strands, viral RNA quantification across varying concentrations, and the resistance to proofreading proteins.

For studies that focused on RdRp enzymatic activity, in silico, in vitro, in vivo, and clinical trial studies alike predicted and validated that existing drugs and compounds can effectively disrupt SARS-CoV-2 viral replication processes by targeting the RdRp protein nsp12. Apart from chloroquine and hydroxychloroquine, the findings were consistent regardless of the method used. Furthermore, mechanistic studies shed light on why certain compounds may demonstrate inhibitory behaviors on the molecular level. While in vitro experiments are the tried and true gold standard for virology, computer simulations largely predicted inhibitory behaviors that were reflected in vitro and in human clinical trials.

One of the key overall themes that repeatedly surfaced through the studies was the importance of RdRp being a highly conserved protein across various viruses, which allows it to be a potential prophylactic or therapeutic agent against SARS-CoV-2, given the structural or mechanistic similarities that can prompt viral replication to cease. While many current COVID-19 vaccines and treatments target proteins on the viral surface, they are not as likely to remain effective against new and emerging variants compared to agents that target highly conserved viral regions, such as RdRp. Over 30% of the studies included in this scoping review explored the efficacy of RdRp inhibition not only of the original Alpha SARS-CoV-2 strain but also of other variants of concern, including Beta, Gamma, Delta, Omicron, VOC-202012/01, and England 2 (England 02/2020/407073). All of these studies, which either solely tested enzymatic activity in vitro/in vivo or explored inhibition through both in silico and in vitro/in vivo methods, confirmed that the RdRp inhibitors were effective against the various strains [27,29,45,47,53,60,67,68,69,74,77,97,99]. Wang et al. (2022) explained that such findings are to be expected when RdRp is the main target instead of surface proteins, such as the spike protein or main protease, because only two amino acid mutations have been discovered in RdRp proteins across all the tested SARS-CoV-2 variants since 2019 [69].

This study has several limitations that impact its internal and external validity. Only published, peer-reviewed articles were included in this study, and emerging data posted on preprint servers, such as BioRxiv or MedRxiv, were not analyzed. Furthermore, articles from only two databases, MEDLINE/PubMed and EMBASE, were represented in this study. Finally, the included articles were dependent on keywords in the abstract and linked MeSH terms on MEDLINE/PubMed; it is possible that relevant articles may have been overlooked due to the stringent inclusion criteria and/or indexing at the time of the literature search. However, the search strategies were based on guidance from a biomedical public health specialist librarian, and three reviewers evaluated the eligible records independently to minimize the likelihood of omitting pertinent articles.

This scoping review sought to explore what is known about the functional implications of the RdRp protein in the context of the COVID-19 pandemic, including studies evaluating the effectiveness of SARS-CoV-2 viral inhibition by existing drugs and compounds. As such, the implications of these results affect not only practice and policy but also future research. In silico, in vitro, in vivo, and human clinical trial data suggest that RdRp inhibitors that were initially developed for other viruses or conditions may be used to disrupt SARS-CoV-2 viral replication and thereby mitigate disease progression. While studies that explored both in vitro and in vivo methods had consistent findings [37,69,73,74] and some clinical trials validated these experimental findings, more research needs to be done to assess their real-world applications, as well as clinical relevance and benefit for adults and children, both prophylactically and therapeutically. Given the highly conserved nature of the RdRp protein, functionality should not be hindered by current or future variants that stem from surface mutations. The potential insights highlighted by these studies, which emphasize targeting the RdRp protein instead of surface proteins, may inform future risk mitigation and treatment strategies against novel SARS-CoV-2 variants.

## Figures and Tables

**Table 1:** Summary of studies evaluating RdRp inhibition and/or mechanism in the context of the COVID-19 pandemic by drug/compound and study design.

Drug/Compound Class Specific Agent (Reference)		RdRp Inhibition				RdRp Mechanism			SARS-CoV-2 Variants *
		In Silico	In Vitro	In Vivo	Human Trial	In Silico	In Vitro	In Vivo	
	**Adenosine-like fungal species**								
Cordycepin (Bibi et al. (2022); Li et al. (2022); Rabie (2022a))		**2**	**1**			**1**			**1**
	**Antibodies**								
Engineered super-Ab to hepatitis C RdRp (Glab-ampai et al. (2022))						**1**	**1**		**1**
Poliovirus vaccine that induces Ab against poliovirus RdRp (Comunale et al. (2021))			**1**						
	**Antifungal Antibiotics**								
Biosynthetic gene cluster of NPP B1 (Park et al. (2022))			**1**						
	**Antimalarials**								
Chloroquine; derivatives (i.e., Hydroxychloroquine) (Gautret et al. (2021); Nimgampalle et al. (2021); Maisonnasse et al. (2020); Yao et al. (2020))		**2**	**2**	**1**	**1**				
	**Antioxidants**								
C60 Fullerene (Hurmach et al. (2021a); Hurmach et al. (2021b))						**2**	**1**		
Quercetin and Theaflavin (Goc et al. (2022))			**1**						**1**
Quercetin and Luteolin (Munafò et al. (2022))		**1**	**1**						
Taroxaz-104 (Rabie (2021))		**1**	**1**						**1**
	**Antiparasitics**								
Suramin (Yin et al. (2021))						**1**	**1**		
	**Antivirals**								
BMS-986094 (developed for hepatitis C) (Jimenez-Guardeño et al. (2022))		**1**	**1**						**1**
Remdesivir (developed for Ebola virus) (Aranda et al. (2022); Choudhury et al. (2020); Koulgi et al. (2020); Nguyen et al. (2020); Pirzada et al. (2021); Stevens et al. (2022); Tchesnokov et al. (2020); Vangeel et al. (2022); Zhang & Zhou (2020))		**1**	**2**			**7**	**2**		**2**
Molnupiravir (developed for influenza virus); derivatives (i.e., 4′-fluorouridine, NHC) (Gordon et al. (2021); Khoo et al. (2023); Sheahan et al. (2020); Sourimant et al. (2022); Vangeel et al. (2022))			**3**	**2**	**1**		**1**		**3**
Nucleoside analogs (i.e., didanosine, fludarabine, vidarabine, favipiravir) (Li et al. (2022); Rabie (2022b); Shinkai et al. (2021); Sirijatuphat et al. (2022); Udwadia et al. (2021))					**3**	**2**	**1**		**1**
Sofosbuvir (developed for Hepatitis C) (Jockusch et al. (2020); Sacramento et al. (2021); Yuan et al. (2021))						**2**	**2**		
Uracil derivatives (used for HIV and Hepatitis C) (Siniavin et al. (2022))		**1**	**1**						**1**
	**Vitamins, Natural Compounds, and Extracts**								
Vitamin B12 (Jimenez-Guardeño et al. (2022); Narayanan & Nair (2020))		**2**	**1**						**1**
Gossypol (used in cancer therapies) (Wang et al. (2022))		**1**	**1**	**1**					**1**
Hypericin (used in cancer therapies) (Matos et al. (2021))		**1**	**1**						
Corilagin (used in cancer therapies) (Li et al. (2021))						**1**	**1**		

* SARS-CoV-2 variants = study tests RdRp inhibition of SARS-CoV-2 variant strain(s) (not Alpha).

## Data Availability

No new data were created or analyzed in this study. Data sharing is not applicable to this article.
